# Intestinal Microbiota Influence Immune Tolerance Post Allogeneic Hematopoietic Cell Transplantation and Intestinal GVHD

**DOI:** 10.3389/fimmu.2018.03179

**Published:** 2019-01-17

**Authors:** Natalie Köhler, Robert Zeiser

**Affiliations:** Department of Hematology, Oncology and Stem Cell Transplantation, Faculty of Medicine, University Medical Center, University of Freiburg, Freiburg, Germany

**Keywords:** GVHD, allo-HCT, microbiota, intestinal inflammation, microbial metabolites, Paneth cells

## Abstract

Under normal conditions our intestines are inhabited by trillions of diverse microorganisms composing the intestinal microbiota, which are mostly non-pathogenic anaerobic commensal bacteria vital for the maintenance of immune homeostasis. The composition and diversity of the intestinal microbiota can be disturbed by various factors including diet, antibiotics, and exposure to intestinal pathogens. Alterations of the intestinal microbiota contributes to many diseases including graft-vs.-host disease (GVHD), a life threatening complication that occurs after allogeneic hematopoietic cell transplantation (allo-HCT) caused by an allogeneic reaction of donor T cells against recipient target tissues. Intestinal GVHD is most difficult to treat and connected to a high mortality. Due to recent advances in high-throughput sequencing technology, composition of the microbiome during allo-HCT has been characterized, and some common patterns have been identified. Metabolites produced by intestinal bacteria were shown to promote intestinal tissue homeostasis and immune tolerance post-allo-HCT. In this review, we discuss the role of the intestinal microbiota and metabolites in the context of acute GVHD. Moreover, novel therapeutic approaches that aim at protecting or regenerating intestinal cell populations will be highlighted.

## Introduction

Allogeneic hematopoietic cell transplantation (allo-HCT) is the only curative therapy option for most acute leukemias. Prior allo-HCT patients undergo a conditioning regimen, consisting of chemotherapy with or without radiotherapy to decrease the number of malignant cells, and prevent rejection of the transplanted hematopoietic donor cells. Subsequently, hematopoietic stem cells (HSCs) from the bone marrow or peripheral blood of an allogeneic donor are infused. In addition to the HSCs, the graft contains allogeneic donor T cells, which can on the one hand attack residual malignant cells, known as graft-vs.-leukemia (GvL) effect, but on the other hand may attack healthy host tissues, resulting in graft-vs.-host disease (GVHD). Although the understanding of the disease pathophysiology has improved significantly in the last decades, acute GVHD is still a major cause of non-relapse morbidity, and mortality post-allo-HCT (1). In particular, patients developing acute GVHD that is refractory to standard steroid therapy are difficult to treat and have a dismal prognosis with only 5–30% overall survival (2–4).

The important influence of intestinal microbiota on immune responses, including post-allo-HCT, becomes more and more recognized and the gut is one of the main targets of acute GVHD. Severe gastrointestinal (GI) GVHD remains a major issue after allo-HCT, since it is difficult to treat and involvement of the GI tract is reported in almost all fatal acute GVHD cases (5). In addition to allogeneic donor T cells, recipient-derived myeloid cells have been shown to participate in GI GVHD development. A number of publications have demonstrated that innate immune cells, including neutrophils and inflammatory monocytes, are recruited to the gut shortly after allo-HCT and contribute to GVHD tissue damage (6–9). The recruitment of neutrophils was dependent on the translocation of bacteria into the intestinal wall and was abrogated in mice housed under germ-free conditions or subjected to antibiotics-based decontamination (6, 10). While in the early phase after allo-HCT antibiotics can reduce the number of transmigrated bacteria which is beneficial, the long-term effects of antibiotics are unfavorable because they limit the microbiota diversity.

## Intestinal Microbiota and GVHD

### Brief Historic Background

The first hint that the intestinal microbiota affect GVHD development dates back to the early 1970s, when first studies in murine models showed that GVHD was reduced and survival was prolonged in antibiotics-treated or germ-free mice undergoing allo-HCT (11–13).

Consequently, efforts were made to translate these insights into the human setting. Clinical trials investigated gut decontamination using antibiotics or laminar-airflow isolation rooms for patients undergoing allo-HCT, in order to minimize contact to microbiota (14–19). However, the study designs were heterogeneous and the outcomes showed inconsistent results. More recent trials even demonstrated that broad spectrum antibiotics increased GVHD-related mortality in allo-HCT patients and mice (20) and the lack of antibiotics that spare commensal bacteria remains a so far unresolved challenge (21). Therefore, to date no standardized protocol for prophylactic and peri-transplant antibiotic treatment has been established as standard of care across transplantation centers.

However, interest in the interplay between the microbiome and inflammatory conditions has been growing again in recent years. Major advances in modern high-throughput sequencing technology made it possible to investigate changes in the composition of the intestinal microflora after allo-HCT in depth and to analyze which bacteria might be detrimental and which may be beneficial with respect to immune tolerance post-allo-HCT.

Under physiological conditions, the intestinal microflora is highly diverse with a domination of anaerobic commensal bacteria, most importantly members of the *Firmicutes* and *Bacteroidetes* phyla (22, 23). During allo-HCT, this diversity is reduced and significant alterations in the microbial composition of the gut can be observed, indicating that the conditioning and/or transplantation induce an intestinal dysbiosis (24–26). Holler et al. demonstrated shifts in the intestinal microbiome after allo-HCT with a predominant increase in the proportion of *Enterococci* (24). This shift was associated with development of GI GVHD. The mean proportion of *Enterococci* was 21% in patients who did not develop GI GVHD as compared to 46% in those that subsequently developed GI GVHD and 74% at the time of active GVHD (24). Moreover, lower intestinal diversity has been shown to be associated with significantly worse mortality outcomes in allo-HCT patients, suggesting that the intestinal microbiota may be an important factor in the success or failure in allo-HCT (25). Looking more specifically at the composition of the microbiota of patients who died vs. patients who survived, greater abundance of γ*-Proteobacteria*, including *Enterobacteriaceae* correlated with increased mortality, whereas greater abundance of *Lachnospiraceae* and *Actinomycetaceae* was associated with favorable outcomes (25).

Since those first innovative studies, a lot of work has been done to investigate how the intestinal microbiota affects immune tolerance post-allo-HCT. A list of preclinical and clinical studies that have analyzed the role of specific bacteria during GVHD pathogenesis can be found in Table 1 and has been reviewed in detail elsewhere (37, 38).

**Table 1:** Summary of studies investigating how microbiota changes affect GVHD (structured by phylum).

**Bacterium**	**Role in GVHD**	**Species**	**References**
**PROTEOBACTERIA**			
*Escherichia coli*	Murine GI GVHD was accompanied by flora shifts toward *E. coli* and this increase was significantly associated with GVHD severity and mortality.	Mouse	Heimesaat et al. (27)
**FIRMICUTES**			
*Enterococcus* spp.	Expansion post-transplantation and association with increased GI GVHD severity in allo-HCT patients.	Human	Holler et al. (24)
	Associated with increased GVHD severity in mice and in patients in three different centers. Aggravation of GVHD in a murine MHC-disparate model.	Human/Mouse	Stein-Thoeringer et al. (28)
*Lactobacillus johnsonii*	GVHD was accompanied by increase in Lactobacillales and decrease in Clostridiales in mice and patients. Ampicillin treatment before allo-HCT resulted in reduced survival in GVHD mouse models. L. johnsonii reintroduction prevented increased GVHD lethality and pathology and prevented *Enterococcus* expansion in mice.	Human/Mouse	Jenq et al. (29)
*Lactobacillus rhamnosus* GG	Oral administration reduced translocation of enteric bacteria and acute GVHD in a murine model.	Mouse	Gerbitz et al. (30)
	Randomized trial of probiotic treatment in 31 allo-HCT recipients. The trial was terminated when interim analysis did not detect an appreciable probiotic-related change in the gut microbiome or incidence of GVHD.	Human	Gorshein et al. (31)
*Lactobacillus plantarum*	Ongoing clinical trial aiming to prevent GVHD by orally-administered *L. plantarum* in children undergoing allo-HCT. Preliminary results demonstrated safety and feasibility.	Human	Ladas et al. (32)
*Clostridiales* spp.	Clinical trial (64 patients, stool analyzed 12 days after BMT) showing that *Blautia* is associated with reduced GVHD-related mortality. Data were confirmed in a 2nd cohort with 51 patients.	Human	Jenq et al. (33)
	Oral gavage with *Clostridia* spp. reduced GVHD severity and mortality in murine mouse models.	Mouse	Mathewson et al. (34)
	Depletion of *Clostridia* spp. was associated with increased GVHD in 15 pediatric allo-HCT patients. Treatment with clinda-mycin depleted *Clostridia* and exacerbated GVHD in mice, while *Clostridia* supplementation reduced murine GVHD severity.	Human/ Mouse	Simms-Waldrip et al. (35)
**BACTEROIDETES**			
*Barnesiella* spp.	*Barnesiella* spp. conferred protection against *Enterococcus* domination in allo-HCT patients and mice.	Human/ Mouse	Ubeda et al. (36)
*Bacteroides/Prevotella* spp.	*Bacteroides/Prevotella* spp. increased during GI GVHD in mice.	Mouse	Heimesaat et al. (27)
**VERRUCOMICROBIA**			
*Akkermansia muciniphila*	Allo-HCT recipients (*n* = 857) as well as GVHD mice treated with broad-spectrum antibiotics showed increased GVHD severity. Imipenem-cilastatin treatment caused destruction of the colonic mucus layer and expansion of *Akkermansia muciniphila* in mice.	Human/Mouse	Shono et al. (20)

In the following, we will highlight the most recent of these findings as well as the latest clinical trials aiming to reduce GVHD by manipulating the intestinal microbiota.

### Recent Developments

Following up on previous studies showing post-transplant monodomination of the gut microbiome with *Enterococcus* spp. in a smaller number of allo-HCT patients (24, 39), these findings were recently confirmed in a large cohort derived from three different centers (28). Monodomination with *Enterococcus* was significantly associated with severe acute GVHD. Moreover, oral administration of *Enterococcus faecalis* following transplantation significantly aggravated acute GVHD in a murine MHC-mismatched model, indicating a causative role for *Enterococcus* spp. in the pathogenesis of acute GVHD (28).

Another study found a significant depletion of anti-inflammatory *Clostridia* spp. (AIC) preceding the development of GVHD in pediatric allo-HCT patients (35). Treatment with anti-anaerobic antibiotics and subsequent depletion of AIC was associated with increased GVHD. These clinical observations were also validated in a murine GVHD model, where clindamycin depleted *Clostridia* and exacerbated disease, whereas oral AIC supplementation attenuated GVHD (35).

In a very recent monocentric study comprising 275 patients, the cumulative incidence of acute GVHD, in particular in the gut, was significantly increased in allo-HCT recipients treated with 4th generation cephalosporins peritransplant (40). A retrospective analysis of 857 allo-HCT recipients by Shono et al. demonstrated that treatment with the broadband antibiotics imipenem-cilastatin or piperacillin-tazobactam was associated with increased GVHD-related mortality at 5 years (20). However, in contrast with the aforementioned study (40), GVHD-related mortality was not correlated with cefepime or aztreonam therapy. Moreover, imipenem-cilastatin treatment caused destruction of the colonic mucus layer in mice and expansion of *Akkermansia muciniphila*, which is a commensal bacterium with mucus-degrading capabilities (41, 42). These data suggest that mucus degradation might aggravate murine GVHD (20).

Based on these recent advances in the field, several clinical trials are currently aiming to reduce GVHD by manipulating the intestinal microbiota. Fecal microbiota transplantation (FMT), also known as stool transplantation, has been used to successfully treat recurrent *Clostridium difficile* infection in non-HCT patients (43). Very recently, first promising data from studies investigating FMT in allo-HCT patients were published (44, 45). A pilot study used FMT from healthy donors to treat eight patients with steroid refractory acute GI GVHD, who have a dismal prognosis (NCT03148743). Following FMT, bacterial diversity and proportion of beneficial bacteria, such as *Bacteroides*, increased and clinical symptoms relieved (44). Another recent pilot trial assessed the effect of third-party oral FMT early after allogeneic HCT (NCT02733744). First studies showed that FMT was feasible and associated with improvements in microbiome diversity in transplant recipients (45). Further studies assessing the safety and efficacy of FMT for GVHD prophylaxis are ongoing (NCT03214289, NCT03359980, NCT03549676, NCT03492502).

## The Effect of Microbial Metabolites on GVHD

**Figure 1:**
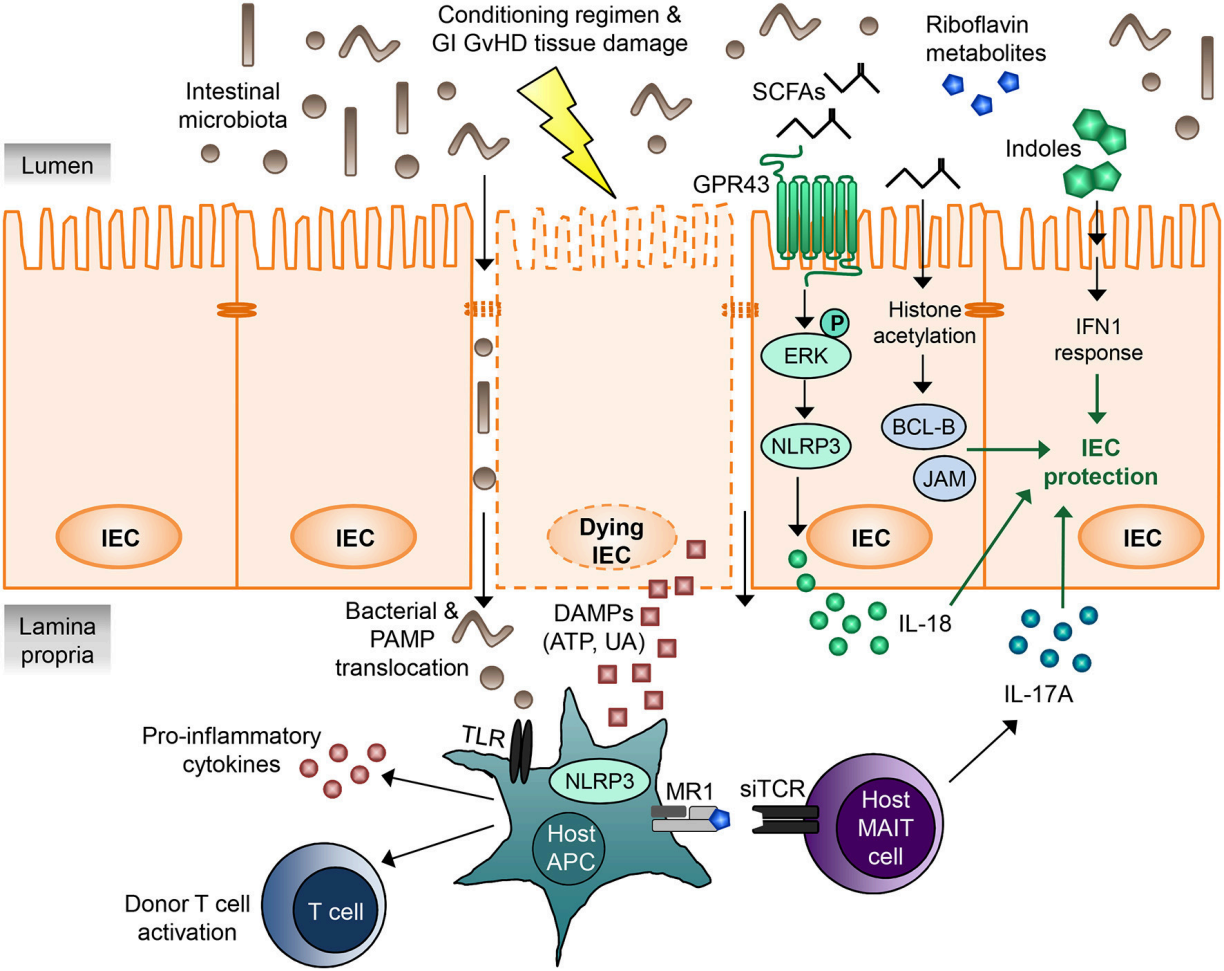
Microbial metabolites regulating gastrointestinal GVHD. IECs are damaged by the cytotoxic conditioning regimen as well as by GI GVHD, leading to disruption of the intestinal barrier. DAMPs released by the dying IECs as well as translocating bacteria and PAMPs activate host APCs via TLRs and the NLRP3 inflammasome, resulting in pro-inflammatory cytokine release, donor T cell activation, and GVHD. Microbial metabolites derived from intestinal microbiota can regulate IEC damage and mitigate GVHD. SCFAs mediate IEC protection via at least two different mechanisms. Firstly, binding of SCFAs to the G-protein-coupled receptor GPR43 on IECs leads to ERK phosphorylation and subsequent NLRP3 inflammasome activation, which promotes IEC integrity and repair by increasing IL-18 secretion. Secondly, the SCFA butyrate acts as a histone deacetylase inhibitor, thereby increasing expression of many different target genes, including anti-apoptotic BCL-B and the junctional protein JAM. This results in decreased IEC apoptosis and increased junctional integrity and hence IEC protection. MAIT cells located in the lamina propria respond to riboflavin metabolite antigens presented on the MHC class I-like molecule MR-1 to secrete large amounts of IL-17A, which enhances intestinal barrier integrity. Indoles and indole derivatives act via type I IFN signaling to protect and repair the mucosal barrier from damage and ameliorate GVHD. The exact molecular mechanisms and involved proteins remain to be elucidated. APC, antigen-presenting cell; DAMP, danger-associated molecular pattern; GI, gastrointestinal; GVHD, graft-vs.-host disease; IEC, intestinal epithelial cell; IFN, interferon; JAM, junctional adhesion molecule; MAIT cell, mucosal-associated invariant T cell; PAMP, pathogen-associated molecular pattern; SCFA, short chain fatty acid; siTCR, semiinvariant T cell receptor; TLR, Toll-like receptor; UA, uric acid.

Microbial metabolites comprise a multitude of different intermediate products and end products of intestinal microbiota metabolism with various functions, yet their role in GVHD pathophysiology is merely starting to be discovered. Very recent publications revealed that one of the mechanisms, how intestinal bacteria can influence immune tolerance post-allo-HCT, is the production of metabolites (Figure 1). Table 2 summarizes studies that have investigated the role of microbial metabolites in epithelial regeneration.

**Table 2:** Summary of studies investigating how microbial metabolites affect intestinal epithelial regeneration.

**Metabolite**	**Effect on intestinal epithelium**	**References**
**AHR LIGANDS**		
AhR ligands	In the intestine, AhR deficiency or lack of AhR ligands reduced intraepithelial lymphocyte numbers and the control of the microbial load, resulting in increased immune activation, and increased vulnerability to epithelial damage.	Li et al. (46)
Indole-3-aldehyde	*Lactobacillus*-derived indole-3-aldehyde induced AhR-dependent transcription of the epithelial cell regenerative factor IL-22 in mice.	Zelante et al. (47)
	Administration of indole-3-aldehyde reduced disease severity, damage of the intestinal epithelium, and transepithelial bacterial translocation in a GVHD mouse model. The effects were mediated through an increased type I interferon response.	Swimm et al. (48)
**SHORT CHAIN FATTY ACIDS (SCFAS)**		
Butyrate	Butyrate added to colonocytes from germfree mice rescued their deficit in mitochondrial respiration and prevented them from undergoing autophagy by acting as an energy source.	Donohoe et al. (49)
	Administration of butyrate improved IEC junctional integrity and reduced clinical scores and mortality in different murine GVHD models.	Mathewson et al. (34)
Butyrate and propionate	Sensing of SCFAs by GPR43 reduced GVHD severity and mortality in mouse models by activating the NLRP3 inflammasome in recipient non-hematopoietic cells via ERK phosphorylation.	Fujiwara et al. (50)
Acetate, butyrate and propionate	SCFAs were important for maintaining intestinal barrier integrity by stimulating MUC-2 production in goblet cells.	Willemsen et al. (51)
	Treatment with SCFAs enhanced IEC migration and promoted wound healing by promoting production of milk fat globulin E8 in mouse and rat IECs *in vitro*.	Bilotta et al. (52)
Acetate	Acetate produced by bifidobacteria protected mice from enteropathogenic infection by improving intestinal defense mediated by IECs.	Fukuda et al. (53)
**BILE ACIDS AND POLYAMINES**		
Bile acids	Lack of the farnesoid X receptor, a receptor for bile acids, caused reduced epithelial barrier function, and increased bacterial translocation in the distal small intestine.	Inagaki et al. (54)
Polyamines	*In vitro* study showing that polyamines enhanced E-cadherin transcription by activating c-Myc, thereby promoting epithelial barrier function.	Liu et al. (55)
**RIBOFLAVIN METABOLITES**		
Riboflavin metabolites	Intestinal MAIT cells responding to microbial riboflavin metabolites produced IL-17A, promoted GI tract integrity and ameliorated intestinal GVHD.	Varelias et al. (56)

Indole and indole derivatives are produced by commensal bacteria using the enzyme tryptophanase and are known to enhance epithelial barrier integrity and to attenuate inflammation (57). Several reports have shown that in the GI tract, bacterial-, and diet-derived indoles engage the aryl hydrocarbon receptor (AhR) and thereby expand innate lymphoid cells (ILC3) and their production of IL-22 (47, 58–60). Specifically, the tryptophan metabolite indole-3-aldehyde produced by intestinal microbiota induces AhR-dependent *Il22* transcription and mucosal immune homeostasis (47). The important role of IL-22 in the protection of intestinal tissue is further discussed below. Very recently, indole and indole derivatives either produced by administered *E. coli* strains or administered exogenously have been shown to strongly reduce GVHD severity, damage of the intestinal epithelium, and transepithelial bacterial translocation, while the GvL effect was not compromised. Mechanistically it was demonstrated that the effects of indole administration were mediated through an increased type I interferon response in an IL-22 independent fashion (48).

Another study focusing on microbiota-derived metabolites could show that the short-chain fatty acid butyrate was significantly reduced in murine intestinal tissue post-allo-HCT resulting in diminished histone acetylation in intestinal epithelial cells (IECs) (34). Daily intragastric administration of butyrate improved IEC junctional integrity and reduced clinical scores and mortality in different murine GVHD models (34). Moreover, butyrate-producing bacteria in the gut were shown to be associated with increased resistance against respiratory viral infection in allo-HCT patients, indicating a favorable role of these bacteria for both immune regulation and prevention of infection after allo-HCT (61). Interestingly, a clinical trial analyzing the role of oral potato-based starch, which is able to increase microbial butyrate production (62), in GVHD prevention is currently recruiting (NCT02763033).

Focusing more on the receptors of bacterial metabolites, the group of Dr. Reddy recently revealed an important regulatory role for GPR43, a G-protein-coupled receptor on IECs recognizing the microbiota-derived short-chain fatty acids, butyrate, and propionate, during GVHD development. Sensing of the microbial metabolites by GPR43 reduced GVHD severity and mortality in murine models by activating the NLRP3 inflammasome in recipient non-hematopoietic cells via ERK phosphorylation. Importantly, GPR43 did not seem to play a role for host and donor hematopoietic cells, since absence of GPR43 did not lead to systemic activation of donor T cells or inflammation (50). While previous reports have already demonstrated a critical role of NLRP3 inflammasome activation in host APCs for the full manifestation of GVHD (63), this study shows that it has the opposite effect in non-hematopoietic IECs. Hence, these data shed new light on the function of NLRP3 in the pathogenesis of GVHD, implicating that the NLRP3 inflammasome can either mitigate or exacerbate GVHD, depending on the involved cell type.

In a recently published study, Varelias et al. demonstrated that recipient mucosal-associated invariant T (MAIT) cells are present in acute GVHD target organs in murine models, including the intestinal lamina propria (56). These cells express semiinvariant T cell receptor repertoires recognizing and responding to microbial riboflavin metabolites. During acute GVHD, MAIT cells generated large amounts of IL-17A, enhanced intestinal barrier function and reduced GI GVHD severity (56). Given the known ability of IL-17 to maintain epithelial cell barrier function (64, 65), the authors conclude that riboflavin metabolite sensing MAIT cells regulate acute GVHD at least partially via IL-17A secretion.

## Strategies Aiming to Protect or Regenerate ISCs and Paneth Cells

Severe GI GVHD remains a major issue after allo-HCT, since it is difficult to treat and involvement of the GI tract is reported in almost all fatal acute GVHD cases (5). Therefore, new therapy approaches aiming for tissue regeneration in GVHD target organs, as opposed to systemic immunosuppression, are promising.

### R-Spondin1 (R-Spo1)

Intestinal stem cells (ISCs) represent important players for both physiological renewal of intestinal cells and tissue regeneration after injury. In a murine model, it was shown that ISCs are damaged during the conditioning regimen and the development of GVHD (66). This could be rescued by treatment with the Wnt agonist R-spondin1 (R-Spo1), which protected against ISC damage after allo-HCT, thereby ameliorating GVHD. In a subsequent study the authors extended their findings, demonstrating that R-Spo1 induces differentiation of ISCs into Paneth cells (67). Paneth cells are known to produce anti-microbial peptides, most importantly alpha defensins, which shape the intestinal antimicrobial flora by mostly targeting non-commensals. Targeting and destruction of Paneth cells during GVHD has already been reported (29, 68, 69). Consistently, by inducing ISC differentiation into Paneth cells, R-Spo1 treatment augmented secretion of alpha defensins, and prevented GVHD-mediated dysbiosis, restoring intestinal homeostasis. The authors suggest that R-Spo1 could inhibit the growth of pathogenic non-commensals, while protecting favorable symbiotic bacteria (67). These findings represent a potential novel strategy to physiologically shape the intestinal microbiome.

### IL-22

IL-22 is a member of the IL-10 cytokine family that plays opposing roles in different autoimmune diseases, being able to drive or regulate disease (70–73). Similarly, both protective and pro-inflammatory roles for IL-22 in GVHD models have been described. On the one hand, IL-22 was recently shown to exacerbate acute GVHD by reducing regulatory T cells in a parent-to-F1 mouse model (74) and to promote cutaneous chronic GVHD (75).

In contrast, Hanash et al. demonstrated that recipient innate lymphoid cell (ILC)-derived IL-22 was able to enhance regeneration of IL-22R-expressing ISCs in a more acute major mismatch GVHD model (76). Host deficiency of IL-22 caused increased epithelial damage, loss of ISCs, and loss of epithelial integrity, resulting in GVHD exacerbation. In a subsequent study, the Hanash group further elucidated the underlying mechanisms, showing that IL-22 increased the growth of murine and human organoids by inducing STAT3 phosphorylation in Lgr5^+^ ISCs (77). Moreover, the authors used two different *in vivo* treatment approaches and showed the feasibility of both recombinant IL-22 and a recombinant human IL-22-dimer/Fc-fusion protein (F-652) for the treatment of murine GVHD. Based on the results from these preclinical studies, a Phase IIa clinical trial investigating F-652 in combination with systemic corticosteroids in patients with grade II-IV lower GI acute GVHD is currently recruiting (NCT0240665).

The described conflicting data of both a regulatory and inflammatory role for IL-22 in GVHD might result from many factors differing between the studies, including kinetics of the model, source of IL-22, presence or absence of other cytokines and pathophysiological differences between acute and chronic GVHD models.

### Regenerating Islet-Derived Protein 3 Alpha (REG3α)

A recent study nicely demonstrated the role of the Paneth cell derived protein Regenerating islet-derived protein 3 alpha (REG3α) to regulate GI acute GVHD (78). REG3α blood levels were strongly upregulated in patients developing severe GI GVHD, which had been shown before (79), and increased blood levels of REG3α in patients with GVHD inversely correlated with Paneth cell numbers. In murine models, the authors demonstrated that absence of REG3γ, which is the mouse homolog of REG3α, exacerbated GVHD without altering the microbial composition in the intestine (78). IL-22 is a known regulator of REG3γ expression (80, 81). In agreement with this, IL-22 administration restored REG3γ production and intestinal epithelial integrity by preventing ISC and Paneth cell apoptosis, resulting in amelioration of GVHD. In *Reg3*γ deficient mice this protection was completely abrogated, emphasizing the important role of REG3γ for gastrointestinal crypt homeostasis (78).

Since host genetics can shape the microbiome, single nucleotide polymorphisms (SNPs) in the genes encoding two of the most abundant Paneth cell antimicrobial peptides REG3α and HD5 were studied (82). Interestingly, SNPs in the gene for HD5 modulated the risk for acute GVHD severity in allo-HCT recipients, potentially by affecting intestinal expression of HD5, which leads to dysbiosis. In contrast, *REG3A* SNPs were not associated with acute GVHD severity in this retrospective study including 350 patients (82).

### Keratinocyte Growth Factor (KGF)

Keratinocyte growth factor (KGF) is a member of the fibroblast growth factor family, regulating differentiation, and proliferation of epithelial cells, including IECs, as well as intestinal stem cells (83, 84). Almost 20 years ago, KGF was already shown to ameliorate GVHD-induced tissue damage in murine models, while preserving GvL effects (85, 86). Based on these preclinical findings two placebo-controlled randomized clinical trials evaluated the efficacy of human recombinant KGF (palifermin) to decrease acute GVHD. Severity of mucositis was reduced by palifermin in a subgroup of patients conditioned with cyclophosphamide and fractionated total-body irradiation in one trial (87). However, in contrast to the findings in murine models, GVHD incidence, severity, and survival were not significantly improved by KGF as compared to placebo in both trials (87, 88). Consistently, a more recent meta-analysis of several clinical studies found no statistically significant difference in oral mucositis and acute GVHD severity in palifermin treated patients compared with those receiving placebo (89).

## Conclusions and Future Perspectives

In the last 10 years it has become evident that a loss of diversity of the intestinal microbiota flora due to reduced food intake, chemotherapy-related damage, and antibiotics promotes the development of GVHD, which changed the dogma that antibiotics-based decontamination of the intestinal tract improves outcome post-allo-HCT. With the discovery that indoles and butyrate promote intestinal homeostasis the connection between antibiotics treatment and unfavorable outcome post-allo-HCT could be explained. Beyond bacterial species that were connected to GVHD, recent studies have also identified fungi and viruses that occurred more frequently in patients with severe GVHD. Based on this improved understanding on how the microbiome and the intestinal tract interact, novel strategies have been developed such as FMT that hold promise to overcome acute GVHD in a majority of patients. Ongoing clinical studies will provide important information on the role of FMT and regenerative strategies.

## Author Contributions

NK and RZ collected literature, discussed the studies, and wrote the manuscript.

### Conflict of Interest Statement

The authors declare that the research was conducted in the absence of any commercial or financial relationships that could be construed as a potential conflict of interest.
